# Maize proteomic responses to separate or overlapping soil drought and two-spotted spider mite stresses

**DOI:** 10.1007/s00425-016-2559-6

**Published:** 2016-06-22

**Authors:** Anna Dworak, Małgorzata Nykiel, Beata Walczak, Anna Miazek, Dagmara Szworst-Łupina, Barbara Zagdańska, Małgorzata Kiełkiewicz

**Affiliations:** 1 Section of Applied Entomology, Faculty of Horticulture, Biotechnology and Landscape Architecture, Warsaw University of Life Sciences-SGGW, 159 Nowoursynowska, 02-776 Warsaw, Poland; 2 Department of Biochemistry, Faculty of Agriculture and Biology, Warsaw University of Life Sciences - SGGW, 159 Nowoursynowska, 02-776 Warsaw, Poland; 3 Institute of Chemistry, Silesian University, 9 Szkolna, 40-006 Katowice, Poland

**Keywords:** Antioxidants, Simultaneous stresses, Stress-related proteins, *Tetranychus urticae*, Water deficiency, *Zea*

## Abstract

**In maize, leaf proteome responses evoked by soil drought applied separately differ from those evoked by mite feeding or both types of stresses occurring simultaneously.**

This study focuses on the involvement of proteomic changes in defence responses of a conventional maize cultivar (Bosman) to the two-spotted spider mite infestation, soil drought and both stresses coexisting for 6 days. Under watering cessation or mite feeding applied separately, the protein carbonylation was not directly linked to the antioxidant enzymes’ activities. Protein carbonylation increased at higher and lower SOD, APX, GR, POX, PPO activities following soil drought and mite feeding, respectively. Combination of these stresses resulted in protein carbonylation decrease despite the increased activity of all antioxidant enzymes (except the CAT). However, maize protein network modification remains unknown upon biotic/abiotic stresses overlapping. Here, using multivariate chemometric methods, 94 leaf protein spots (out of 358 considered; 2-DE) were identified (LC–MS/MS) as differentiating the studied treatments. Only 43 of them had individual discrimination power. The soil drought increased abundance of leaf proteins related mainly to photosynthesis, carbohydrate metabolism, defence (molecular chaperons) and protection. On the contrary, mite feeding decreased the abundance of photosynthesis related proteins and enhanced the abundance of proteins protecting the mite-infested leaf against photoinhibition. The drought and mites occurring simultaneously increased abundance of proteins that may improve the efficiency of carbon fixation, as well as carbohydrate and amino acid metabolism. Furthermore, increased abundance of the Rubisco large subunit-binding protein (subunit β), fructose-bisphosphate aldolase and mitochondrial precursor of Mn-SOD and decreased abundance of the glycolysis-related enzymes in the mite-free leaf (in the vicinity of mite-infested leaf) illustrate the involvement of these proteins in systemic maize response to mite feeding.

**Electronic supplementary material:**

The online version of this article (doi:10.1007/s00425-016-2559-6) contains supplementary material, which is available to authorized users.

## Introduction

Under field conditions, crop plants are exposed to many unavoidable environmental fluctuations (e.g., soil water shortage, flooding, extreme temperatures, salinity, pathogen infection, arthropod herbivore attack). Most of the stress factors increase formation/accumulation of reactive oxygen species (ROS) that change cellular redox metabolism (Baxter et al. 2014). Major producers of ROS are electron transport chains in the chloroplast and mitochondria as well as apoplastic peroxidases and membrane bound NADPH-oxidases. To control ROS generation, plants engage an antioxidant defence system consisting of nonenzymatic antioxidants and ROS scavenging enzymes. The capabilities of ROS-scavengers are essential for the effectiveness of mechanisms protecting plants against ROS overabundance due to biotic/abiotic environmental factors (Foyer and Noctor 2011). At a low dosage, ROS act as second messengers in hormone signalling, coordinately regulating plant stress tolerance, while they cause oxidative damage when the level of ROS is overabundant (Foyer and Noctor 2011; Xia et al. 2015).

Although ROS involvement in plant stress tolerance differs from one stress to another, there is no doubt that ROS formation is required for both local and systemic signalling (Baxter et al. 2014; Xia et al. 2015 and rfs therein) and for activating stress response proteins, such as protein kinases, transcription factors, antioxidant enzymes and/or pathogenesis-related proteins (Atkinson and Urwin 2012). Thus, upon stress-related conditions, proteins appear to be the most frequently targeted by oxidative species. The most prevalent way of oxidatively modified protein formation is either oxidation of sulphur-containing residues of amino acids or oxidation of amino acid side chains to carbonyl derivatives (Levine 2002). On the other hand, redox regulation of proteins is required for activating efficient responses diminishing the negative effects on plant growth, development and productivity (Suzuki et al. 2014; Xia et al. 2015).

Much less is known about the metabolic background of plant responses to coexisting abiotic and biotic stresses, and the issue has not been fully examined yet (Prasch and Sonnewald 2015). However, recent evidence reveals that the effects of a joint action of two or more stresses differ from the effects of those occurring independently (Atkinson et al. 2013; Suzuki et al. 2014; Prasch and Sonnewald 2015). Furthermore, plants exposed to one type of stress develop resistance to other concurrently occurring stresses, and therefore, cross-tolerance to drought, cold, salinity and/or heat is a common phenomenon (Atkinson and Urwin 2012; Suzuki et al. 2014; Prasch and Sonnewald 2015). For example, gene expression induced by low temperature is interrelated with the level of tissue dehydration tolerance, and thus, plant acclimation to cold or frost promotes the development of tolerance to a number of diverse environmental stresses (Grudkowska and Zagdańska 2010). In contrast, the combined drought and heat stresses had detrimental effect on the growth and productivity of barley and sorghum and it was much more pronounced than the one of the same stresses applied separately (Atkinson and Urwin 2012 and rfs therein; Rollins et al. 2013; Suzuki et al. 2014). Drought have been found to increase or decrease plant defence responses to insect herbivores due to hormonal signalling cross talk (Nguyen et al. 2016 and rfs therein).

Maize (*Zea mays* L.), the third agricultural crop worldwide and one of the most commonly cultivated cereals in Europe, is frequently exposed to soil water deficiency (Benešová et al. 2012) accompanied by the occurrence of a broad spectrum of arthropod herbivores (Meissle et al. 2013). In maize field crops, the European corn borer (ECB, *Ostrinia nubilalis*; Lepidoptera), aphid species (*Rapalosiphum padi*, *Sitobion avenae*, *Metopolophium dirhodum*; Hemiptera) and the Western corn rootworm (*Diabrotica virgifera virgifera*; Coleoptera) are the dominating insect herbivores, albeit many other herbivorous arthropods including the two-spotted spider mite, (*Tetranychus urticae* Koch, 1836; Acari, Prostigmata, *Tetranychidae*) frequently inhabit the crop (Cullen and Schramm 2009). The two-spotted spider mite is a generalist with a piercing-sucking mode of feeding. At the site of mite feeding, saliva injection and chelicera mechanical damage trigger cytological/metabolic changes (local responses) that may systemically impact both leaf tissues in the vicinity of mite-infested sites and other leaves within the mite-infested plant (Gawrońska and Kiełkiewicz 1999; Kielkiewicz 1999; Świątek et al. 2014). It may be controlled through JA-signalling (Zhurov et al. 2014) and/or by abscisic acid (ABA) or ethylene involvement (Gawrońska and Kiełkiewicz 1999; Kielkiewicz 2002). The mite-pest’s outbreaks in the field are linked to prolonged hot weather and soil drought conditions (Cullen and Schramm 2009). However, the combined effect of soil drought and mites on plant fitness and tolerance has not yet been fully explained.

Recent studies have revealed that the type of stress such as soil drought or two-spotted spider mite infestation applied separately determined the response of key antioxidant maize enzymes (Świątek et al. 2014). Therefore, the question arises whether the combination of soil drought and mite stresses intensifies oxidative processes or induces distinct responses. To gain more insight into maize responses to co-occurring or separate stresses (drought and mite), the activity of ROS scavenging enzymes and their impact on the level of oxidized proteins as well as the leaf proteome profile were analysed and compared. We used two-dimensional polyacrylamide gel electrophoresis (2-DE) and liquid chromatography–tandem mass spectrometry (LC–MS/MS) as tools for searching stress-related proteins that may contribute to maize tolerance. The conventional maize cultivar (Bosman), before reproductive growth stage (V_11_), was chosen as plant material. Our results provide new evidence that jointly applied soil drought and mite-pest infestation lead to unique changes in the mature maize leaf proteome, differing from those caused by individual stress action, thus contributing to the knowledge on the C4 monocot responses to overlapping environmental stresses.

## Materials and methods

### Plant material and experimental setup

Plants of maize (*Zea mays* L. cv. Bosman, Hodowla Roślin Smolice Sp. z o.o. Grupa IHAR, Poland) were grown in individual pots filled with peat substrate under greenhouse conditions. Six-week-old plants at the eleven-leaf-stage (V_11_) were either subjected to a two-spotted spider mite feeding, soil drought evoked by cessation of watering, or a combination of mite infestation and soil drought stresses for 6 days. The control plants, watered twice a day, were free of mites. The middle part of the maize leaf 8 (fully expanded) of the plants that were subjected to mite infestation was artificially colonised by fifty females (for details see Świątek et al. 2014). The mites were collected from a synchronized lab population continuously reared on bean plants at day/night temperature of 24/18 °C, at 16/8 h photoperiod. Mite-infested leaves were not overcrowded, and the source of food was sufficient to keep the mite females settled in place. After 6 days, leaves from the control and stress-treated plants were excised for further analyses. Additionally, the leaf 9, free of mites (‘noninfested’ from mite-infested plant) might receive a signal from ‘mite-infested leaf 8 and the respective control leaf were collected. The relative water content (RWC) in each leaf was expressed as: RWC (%) = (FW − DW)/(SW − DW), where FW means the leaf fresh weight, DW—the leaf dry weight, 105 °C; SW—the leaf saturated weight (Barrs 1968).

Two series of independent experiments were carried out and six leaf samples collected from six plants were divided into six biological replicates and used for measuring enzyme activity, protein oxidation and protein profiles.

### Antioxidant enzyme activity measurements

The activity of superoxide dismutase (SOD, EC 1.15.1.1) was measured on the basis of reduction of nitroblue tetrazolium (NBT) at 560 nm (Fridovich 1986). The enzyme extract was prepared from leaf tissue (1 g FW) grounded in liquid nitrogen and extracted in a 5 ml pre-cooled extraction buffer (50 mM Tris–HCl pH 7.5) containing 1 % (w/v) insoluble polyvinylpyrrolidone (PVP). The homogenate was centrifuged at 20,000*g* (4 °C; 20 min) and the supernatant was directly used for the enzyme assays. The reaction mixture contained 12.48 μM riboflavin, 13 mM methionine, 75 μM NBT in a 0.1 M phosphate buffer pH 7.8 and 50 μl of crude enzyme extract in the total volume of 2.5 ml. One unit of SOD activity was expressed as enzyme activity inhibiting the photoreduction of NBT to blue formazan by 50 %.

The ascorbate peroxidase (APX, EC 1.11.1.11) was extracted and assayed as described by (Nakano and Asada 1981). The enzyme extract was prepared from leaf tissue (1 g FW) grounded in liquid nitrogen. Then 5 ml 50 mM phosphate buffer pH 7.0 containing 1 % (w/v) insoluble PVP, 0.1 mM EDTA and 2 mM ascorbate was added. The homogenate was centrifuged at 15,000*g* (4 °C; 20 min). The reaction mixture containing 0.1 mM H_2_O_2_ was incubated together with the enzyme extract (30 µl) in the total volume of 1 ml. The change in absorbance at 290 nm was recorded every 10 s for 3 min. The APX activity was calculated using an extinction coefficient for ascorbate (2.8 mM^−1^ cm^−1^) and expressed as units per mg of protein, where one unit of APX activity was expressed as ascorbate µmoles oxidized per minute.

The catalase (CAT, EC 1.11.1.6) activity was measured by determining the degree of H_2_O_2_ decomposition at 240 nm for 2 min (Beers and Sizer 1952). An enzyme extract was prepared from leaf tissue (1 g FW) grounded in liquid nitrogen and extracted in a 5 ml pre-cooled extraction buffer (50 mM Tris–HCl pH 7.5) containing 1 % (w/v) insoluble PVP. The homogenate was centrifuged at 20,000*g* (4 °C; 20 min) and supernatant was directly used for the enzyme assays. The reaction mixture contained 20.4 mM H_2_O_2_ in a 50 mM potassium phosphate buffer pH 7.0 (1 ml), 100 μl of crude enzyme extract (100 μl) and deionized water (1.9 ml). One unit of CAT activity was expressed as H_2_O_2_ μmoles (39.4 mM^−1^ cm^−1^) removed per minute.

The glutathione reductase (GR, EC 1.6.4.2) activity in crude extract was assayed by monitoring the levels of NADPH glutathione-dependent oxidation at 340 nm (Foyer and Halliwell 1976). Leaf FW (0.1 g) was pulverised in liquid nitrogen and extracted with 2 ml 50 mM phosphate buffer pH 7.5 containing 1 mM EDTA, 10 mM sodium ascorbate and 0.2 g insoluble PVP. The homogenate was centrifuged at 15,000*g* (4 °C; 10 min). The assay mixture contained 50 mM phosphate buffer pH 7.5, 0.15 mM NADPH, 10 mM glutathione disulphide (GSSG) and the crude enzyme extract (0.l ml) in the total reaction volume of 1 ml. GR activity was expressed as NADPH nmol per mg of protein.

Using guaiacol as a substrate, the guaiacol peroxidase (POX, EC 1.11.1.7) activity was assayed. The enzyme extract was prepared from leaf tissue (1 g FW) grounded in liquid nitrogen and extracted in a 5 ml 50 mM Tris–HCl pH 7.5 containing 1 % (w/v) insoluble PVP. The homogenate was centrifuged at 20,000*g* (4 °C; 20 min) and supernatant was directly used for enzyme assays. The reaction medium consisted of 4.5 mM guaiacol (0.5 ml) and 4.9 mM H_2_O_2_ (0.5 ml) in a 50 mM acetate buffer pH 5.6 (0.99 ml). The reaction was initiated by adding 10 μl of crude enzyme extract (Patykowski et al. 2007) and an increase in absorbance at 470 nm was monitored for 4 min. The POX activity was expressed as units per mg of protein where one unit of POX activity was expressed as guaiacol μmoles (26.6 mM^−1^ cm^−1^) oxidized per minute.

The polyphenol oxidase (PPO, EC 1.14.18.1) was extracted and assayed as described by Zauberman et al. (1991) with some modifications. Leaf tissue (0.1 g FW) was pulverised in liquid nitrogen and extracted in a 2 ml 50 mM phosphate buffer pH 6.2 containing 50 mM EDTA. The homogenate was centrifuged at 15,000*g* (4 °C; 10 min). The reaction mixture consisted of a 50 mM phosphate buffer pH 6.2, 50 mM pyrogallol and the enzyme extract (0.1 ml) in the total volume of 1 ml. The conversion of pyrogallol to purpurogallin was measured at 420 nm. The PPO activity was expressed as units per mg of protein where one unit of the enzyme activity was expressed as purpurogallin μmoles produced per minute.

The soluble protein content in leaf extracts was quantified using the Bradford (1976) method with bovine serum albumin (BSA) as a standard.

### Protein oxidation measurement

The concentration of the derivatized carbonyl group of oxidized proteins in the presence of 2,4-dinitrophenylhydrazine (DNPH) was determined using the method of Levine et al. (1994). Briefly, maize leaf sample proteins were extracted in a 100 mM phosphate buffer pH 7.8 containing 1 mM EDTA, 2 mM PMSF and 1 µM pepstatin. Aliquot extracts (0.1 ml) were incubated with 10 mM DNPH or 2.5 M HCl in darkness for 1 h (control). The proteins were precipitated with 20 % trichloroacteic acid (TCA) and after 10 min centrifuged at 12,000*g* for 10 min. The protein pellet was washed with ethanol and ethyl acetate (1:1; v/v) three times and dissolved in 6 M guanidine hydrochloride in a 50 mM potassium phosphate buffer pH 2.36. The absorbance was measured at 370 nm. The carbonyl content was assessed using an extinction coefficient of hydrazone (22,000 M^−1^ cm^−1^) and expressed as C=O nmol per mg of protein.

### Statistical analysis

The variance analysis (one-way ANOVA) at the 95 % confidence level was used to assess differences in the activity of the leaf antioxidant enzymes as well as in the content of oxidized and total proteins. The Tukey’s honestly significant difference (HSD) test and the nonparametric Kruskal–Wallis test were performed to separate means and medians, respectively. The significance level was set to 0.05. The data are presented as the mean ± SD. All statistical analyses were performed using Statistica 10.0 software.

### Leaf sample proteomic analysis setup

To extract leaf proteins, the leaf samples (0.3 g) grounded in liquid nitrogen were resuspended in 2.0 ml 10 % TCA, dissolved in cold acetone, vortexed for 30 s and centrifuged at 10,000*g* (4 °C; 15 min). The fine powder was rinsed with cold 10 % TCA in acetone until the supernatant was colourless. The pellet was washed with 0.1 M ammonium acetate dissolved in 80 % methanol and with cold 80 % acetone. The pellet was vortexed, centrifuged (as above), dried and resuspended in a 0.8 ml phenol and 0.8 ml dense SDS buffer (30 % sucrose, 2 % SDS, 0.1 M Tris–HCl, pH 8.0, 5 % 2-mercaptoethanol). The mixture was vortexed for 3 min. and the phenol phase was separated by centrifugation at 10,000*g* for 30 min. The upper phenol phase (0.4 ml) was mixed with at least five volumes of cold methanol and 0.1 M ammonium acetate and the mixture was stored at −20 °C for 30 min. The precipitated proteins were dried and dissolved at 25 °C for 16 h in a 2-DE rehydration solution (7 M urea, 2 M thiourea, 4 % w/v CHAPS, 2 % v/v IPG buffer and 20 mM DTT).

### Two-dimensional IEF/SDS–PAGE and protein staining

Equal amounts of the extracted proteins (150 µg) were separated by two-dimensional polyacrylamide gel electrophoresis (2-DE) as described by the Bio-Rad protein assay (Bio-Rad Laboratories). In the first dimension, IPG strips (Bio-Rad), each 11-cm long, were used. The pH was between 4 and 7. The isoelectric focusing (IEF) was performed using PROTEAN IEF Cell (Bio-Rad). The electrophoresis was initiated at 250 V for 20 min, followed by 8000 V for 2.5 h, and it was continued until reaching 20,000 Vh. The strips were equilibrated for 15 min in slow agitation in a Tris–HCl solution (75 mM), pH 8.8, containing 2 % w/v SDS, 29, 3 % v/v glycerol, 6 M urea and 100 mM DTT, and subsequently in Tris–HCl (50 mM) pH 6.8 containing 2 % w/v SDS, 29, 30 % v/v glycerol, 6 M urea and 135 mM IAA. After IEF, the proteins were separated by SDS-PAGE in the second dimension using 11 % polyacrylamide gels. The gels were stained by the colloidal Coomassie G-250 method and scanned with the ImageScanner III (GE Healthcare). Six gels in two technical replications were run for each treatment.

### Gel image pre-processing and proteome profile evaluation

Individual gel images require intense pre-processing prior to further data evaluation. In this study, the images were background corrected using the rolling ball method and warped to the selected standard (gel 2 from control leaf 8) (using the Fuzzy Warping approach (Daszykowski et al. 2007) (Fig. S1a–b). Normalized individual images were used to generate the mean image to detect spots and to construct the binary mask (Fig. S2a–c). A comparison of proteomic fingerprints was performed between control class [leaf 8; C(8)] and the class representing stress effect such as mite infestation [Tu + (8)], soil drought [D + (8)], the combination of mite infestation and soil drought stresses [Tu + D(8)], as well as between class [C(9)] (control leaf 9 above leaf 8) and the class representing the indirect mite feeding effect on leaf 9 [Tu − (9)].

After pre-processing the gel images, a variance analysis was performed to test, if the compared classes of samples differed significantly. The variance analysis was performed at both, spot and pixel levels. PERMANOVA was the method of choice for variance analysis (Zerzucha et al. 2012). The randomization test was repeated 10,000 times and the significance level was set to 0.05 (Table 1). The identification of significant features (spots or pixels) was made using the uninformative variable elimination—partial least squares (UVE-PLS) (Zerzucha et al. 2012). Features selection was cross model validated; and depending on how frequently individual features were selected, a final set of the significant ones was built. The final set of significant features contains the ones which were selected in most cases (more than 50 %) (Table 2). The exploratory analysis of studied data was performed by a principal component analysis (PCA) followed by a hierarchical cluster analysis (HC) with the Euclidean distance as a similarity measure and Ward’s linkage method.

**Table 1:** Variance analysis (PERMANOVA) performed at spots and pixel levels

Classes compared	Spots (358)		Pixels (17,616)	
	*F*	*P*	*F*	*P*
C(8) and Tu + (8)	1.3650	0.36	1.7077	0.03*
C(8) and D + (8)	4.7774	0.01*	2.2643	0.00*
C(8) and Tu + D(8)	5.2811	0.00*	2.6821	0.00*
C(8) and C(9)	3.7609	0.05*	2.3190	0.00*
C(9) and Tu − (9)	2.9037	0.06	2.8127	0.00*
Tu + (8) and Tu − (9)	4.4968	0.00*	1.7687	0.00*

Randomization test was performed 10,000 times

* Statistically significant differences (at significance level 0.05)

*F* denotes the value of the *F* test, and *P* refers to the calculated significance level

**Table 2:** The calculated significance values (*P*) for protein spots differentially expressed when compared individually within the studied classes by the multivariate analysis (at *P* < 0.05)

Protein spot number and *P* value for classes compared					
C(8) and Tu + (8)	C(8) and D + (8)	C(8) and Tu + D(8)	C(8) and C(9)	C(9) and Tu − (9)	Tu + (8) and Tu − (9)
2/0.0396	1/0.0013	3/0.0036	1/0.0069	1/0.0069	1/0.0142
10/0.0080	3/0.0314	4/0.0455	2/0.0004	2/0.0084	3/0.0361
11/0.0289	4/0.0001	5/0.0111	5/0.0286	5/0.0004	5/0.0400
12/0.0357	5/0.0015	6/0.0114	10/0.0041	6/0.0286	6/0.0202
	8/0.0077	8/0.0058		13/0.0041	8/0.0034
	11/0.0003	13/0.0081			
	12/0.0440	14/0.0122			
	13/0.0000	15/0.0337			
	14/0.0001	16/0.0125			
	17/0.0000	23/0.0020			
	18/0.0000	24/0.0143			
	20/0.0021	26/0.0226			
	21/0.0199				
4^+^	13^+^	12^+^	4^+^	5^+^	5^+^
12^++^	22^++^	26^++^	12^++^	14^++^	8^++^

+ Indicates the total number of protein spots significantly different when compared individually

++ Indicates the total number of significant spots for the studied class pairs as a result of multivariate approach (for details see Figs. 4a–c, 5a–c)

### Protein identification by LC–MS/MS

Selected protein spots were identified by liquid chromatography–tandem mass spectrometry (LC–MS/MS; nanoAcquity UPLC and Orbitrap type mass spectrometer) at the Mass Spectrometry Lab of the Institute of Biochemistry and Biophysics of Polish Academy of Sciences (Warsaw, Poland). Prior to the analysis, the excised gel slices were subjected to the standard procedure of in-gel trypsin digestion, during which the proteins were reduced with 100 mM DTT at 56 °C for 30 min, alkylated with iodoacetamide at darkroom temperature for 45 min and digested overnight with 10 ng µl^−1^ trypsin. The peptides were eluted from the gel with water solution of 0.1 % trifluoroactetic acid (TFA) and 2 % acetonitrile (ACN). The resulting peptide mixtures were applied to the RP-18 pre-column (Waters, Milford, MA, USA), using water containing 0.1 % formic acid (FA) as a mobile phase, and then transferred to the nano-HPLC RP-18 column (internal diameter 75 μM, Waters) using the ACN gradient (0–30 % ACN in 40 min) in the presence of 0.1 % FA at a flow rate of 250 nl min^−1^. The column outlet was coupled directly to the ion source of Orbitrap Velos mass spectrometer (Thermo) working in the regime of data-dependent MS to MS/MS switch. A blank run preceded each analysis to ensure that the previous samples had not been the cause of cross-contamination.

### Mass spectrometry data analysis

After pre-processing the raw data with Mascot Distiller software (version 2.3, Matrix Science, London, UK), obtained peak lists were used to search the nonredundant protein database of the National Centre for Biotechnology Information (NCBI-NR) (23919380 sequences; 8216485116 residues) using the Mascot search engine (version 2.4, 8-processors onsite license) (Matrix Science) with the following search parameters: taxonomy restriction—Viridiplantae (Green Plants, 1249273 sequences), enzyme specificity—trypsin, permitted number of missed cleavages—1, fixed modification—carbamidomethylation (C), variable modifications—carboxymethyl (K), oxidation (M), protein mass—unrestricted, peptide mass tolerance—±30 ppm, fragment mass tolerance—±0.6 Da. Only the peptides over the Mascot-defined expectation value of 0.05 were considered positive identifications. Data concerning the results of LC–MS/MS are shown in Table 3. Detailed technical information is presented in Table S1 and https://dl.dropboxusercontent.com/u/24272155/widma.zip.

**Table 3:** Identified protein spots differentiating the studied classes: C(8) and Tu + (8), C(8) and D + (8), C(8) and Tu + D(8), C(8) and C(9), C(9) and Tu − (9) as well as Tu + (8) and Tu − (9) in multivariate analysis at *P* ≤ 0.05 (see Table 2)

Protein spot		Protein name (against NBCI database)	Accession	Score	MW/pIt (kDa 10^3^)	Matches	Sequences	Coverage	Biological relevance
No.	**↓/↑**								
C(8) and Tu + (8)									
2	**↓**	Ribulose-1,5-bisphosphate carboxylase/oxygenase (RuBisCO) partial [*Sorghastrum nutans*]	gi|375493221	2437	49.92/6.44	103 (103)	17 (17)	60	Photosynthesis related
10	**↓**	Putative TCP-1/cpn60 chaperonin family protein partial [*Zea mays*] (cpn60)	gi|413942615	1459	35.72/5.09	40 (40)	18 (18)	60	Chaperone protein
11	**↑**	Oxygen evolving enhancer protein3 containing protein [*Zea mays*] (OEE3)	gi|195609634	1668	25.91/7.66	52 (52)	8 (8)	43	Photosynthesis related
12	**↑**	Heat shock cognate 70 kDa protein2 [*Zea mays*] (HSC70)	gi|293334615	1184	71.52/5.13	36 (36)	24 (24)	41	Chaperone protein
C(8) and D + (8)									
1	**↑**	RuBisCO, large subunit, partial (chloroplast) [*Campanula trache*]	gi|253992223	451	50.52/6.46	20 (20)	9 (9)	15	Photosynthesis related
3	**↑**	RuBisCO, small subunit2 [*Zea mays*]	gi|226532904	741	18.19/8.19	28 (28)	9 (9)	64	Photosynthesis related
4	**↓**	Drought-inducible 22 kDa protein [*Saccharum officinarum*]	gi|15667623	174	15.92/7.78	5 (5)	4 (4)	35	Stress responsive
5	**↓**	Plastid ADP-glucose pyrophosphorylase large subunit (AGPase) [*Zea mays*]	gi|162460455	1203	55.50/8.57	26 (26)	9 (9)	24	Starch biosynthesis
8	**↓**	Chloroplast protein synthesis2 [*Zea mays*] (cps2)	gi|413945149	501	63.26/5.06	6 (6)	5 (5)	13	Photosynthesis related
11	**↑**	Glyoxylase1 [*Zea mays*]	gi|162461576	949	32.45/5.39	30 (30)	13 (13)	53	Glyoxalase system
12	**↑**	Unknown [*Zea mays*]	gi|194688752	4449	47.67/5.95	133 (133)	19 (19)	51	Not identified
13	**↑**	17.5 kDa class II heat shock protein [*Zea mays*]	gi|195639038	878	17.80/5.17	33 (33)	4 (4)	32	Heat shock protein
14	**↑**	LOC100192117 [*Zea mays*] (PR-10)	gi|212275926	1466	17.05/5.38	41 (41)	12 (12)	86	Pathogenesis-related protein
17	**↑**	cpn60	gi|413942615	1459	35.73/5.09	40 (40)	18 (18)	60	Chaperone protein
18	**↓**	Unknown [*Zea mays*]	gi|223948025	2695	61.97/5.42	71 (71)	28 (28)	56	Not identified
20	**↑**	NADP-malic enzyme [*Zea mays*] (NADP-ME)	gi|30575690	822	70.41/6.20	23 (23)	6 (6)	29	Photosynthesis related
21	**↓**	RuBisCO large subunit-binding protein subunit α, LOC100281701 [*Zea mays*]	gi|226493235	4487	61.1/5.20	107 (107)	21 (21)	64	Chaperone protein
C(8) and Tu + D(8)									
23	**↑**	Phosphoenolpyruvate carboxylase (PEPC) [*Zea mays*]	gi|27764449	389	109.85/5.7	21 (21)	19 (19)	22	Photosynthesis related
3	**↑**	Pyruvate, orthophosphate dikinase (PPDK) [*Zea mays*]	gi|168586	1589	103.36/5.7	41 (41)	18 (18)	27	Photosynthesis related
8	**↓**	Peptidyl-prolyl *cis*–*trans* isomerase family protein isoform1 [*Zea mays*] (PPIases)	gi|212723348	309	35.3/8.66	9 (9)	8 (8)	29	Chaperone protein
13	**↓**	cps2	gi|413945149	501	63.26/5.06	6 (6)	5 (5)	13	Photosynthesis related
5	**↑**	β-d-glucosidase precursor [*Zea mays*]	gi|343227637	1895	63.46/6.75	64 (64)	18 (18)	30	O-glycosyl compounds hydrolysis
6	**↑**	Drought-inducible 22 kDa protein [*Saccharum officinarum*]	gi|15667623	174	15.92/5.78	5 (5)	4 (4)	35	Stress responsive
14	**↑**	Aspartate aminotransferase (AAT) [*Zea mays*]	gi|226508814	379	50.55/8.15	12 (12)	11 (11)	24	Amino acids biosynthesis
16	**↑**	PPDK [*Zea mays*]	gi|168586	5109	103.3/5.71	149 (149)	30 (30)	42	Photosynthesis related
24	**↑**	Cytosolic PPDK [*Zea mays* subsp. *mays*]	gi|238928442	1949	96.43/5.42	46 (46)	11 (11)	34	Photosynthesis related
26	**↑**	Predicted stromal 70 kDa heat shock-related protein chloroplastic-like [*Brachypodium distachyon*]	gi|357134135	1194	73.20/5.04	37 (37)	15 (15)	25	Heat shock protein
15	**↑**	Unknown [*Zea mays*]	gi|223949895	3742	41.47/4.91	88 (88)	21 (21)	57	Not identified
4	**↓**	Unknown [*Zea mays*]	gi|223949895	692	41.47/4.91	11 (11)	5 (5)	16	Not identified
C(8) and C(9)									
2	**↑**	ATP synthase CF1 α subunit [*Chasmanthium latifolium*] (atpA)	gi|307697215	1937	55.69/5.73	74 (74)	21 (21)	44	Energy metabolism
10	**↓**	PPDK [*Zea mays*]	gi|168586	4547	103.36/5.7	138 (138)	32 (32)	46	Photosynthesis related
5	**↓**	Fructose-bisphosphate aldolase [*Zea mays*]	gi|195634659	3949	41.92/7.63	150 (150)	18 (18)	49	Glycolysis/gluconeogenesis
1	**↓**	Glyceraldehyde-3-phosphate dehydrogenase (GAPDH) cytosolic2 [*Zea mays*]	gi|162461501	1110	36.63/6.41	36 (36)	13 (13)	42	Glycolysis
C(9) and Tu − (9)									
5	**↑**	atpA	gi|307697215	1937	55.69/5.73	74 (74)	21 (21)	44	Energy metabolism
13	**↓**	PPDK [*Zea mays*]	gi|168586	4317	103.36/5.7	135 (135)	36 (36)	47	Photosynthesis related
1	**↓**	GAPDH cytosolic2	gi|162461501	1110	36.63/6.41	36 (36)	13 (13)	42	Glycolysis
2	**↓**	GAPDH partial [*Zea mays*]	gi|293889	1064	26.49/6.25	33 (33)	12 (12)	61	Glycolysis
6	**↓**	Fructose-bisphosphate aldolase [*Zea mays*]	gi|195634659	3949	41.92/7.63	150 (150)	18 (18)	49	Glycolysis/gluconeogenesis
Tu + (8) and Tu − (9)									
1	**↓**	GAPDH cytosolic2 [*Zea mays*]	gi|162461501	1110	36633	36 (36)	13 (13)	42	Glycolysis
3	**↓**	Fructose-bisphosphate aldolase [*Zea mays*]	gi|195634659	518	41.92/7.63	16 (16)	11 (11)	36	Glycolysis/gluconeogenesis
5	**↑**	Superoxide dismutase [Mn] 3.4 mitochondrial precursor [*Zea mays*]	gi|212722004	367	25.21/6.71	15 (15)	7 (7)	38	Antioxidant activity
6	**↑**	RuBisCO large subunit-binding protein subunit β [*Zea mays*]	gi|195630027	942	64.4/5.81	26 (26)	16 (16)	29	Chaperone protein
8	**↑**	Fructose-bisphosphate aldolase [*Zea mays*]	gi|195634659	1277	41.92/7.63	36 (36)	12 (12)	40	Glycolysis/gluconeogenesis

Upward arrow (↑) indicates up-regulating effect

Downward arrow (↓) indicates down-regulating effect

## Results

### Antioxidant enzyme response

**Fig. 1:**
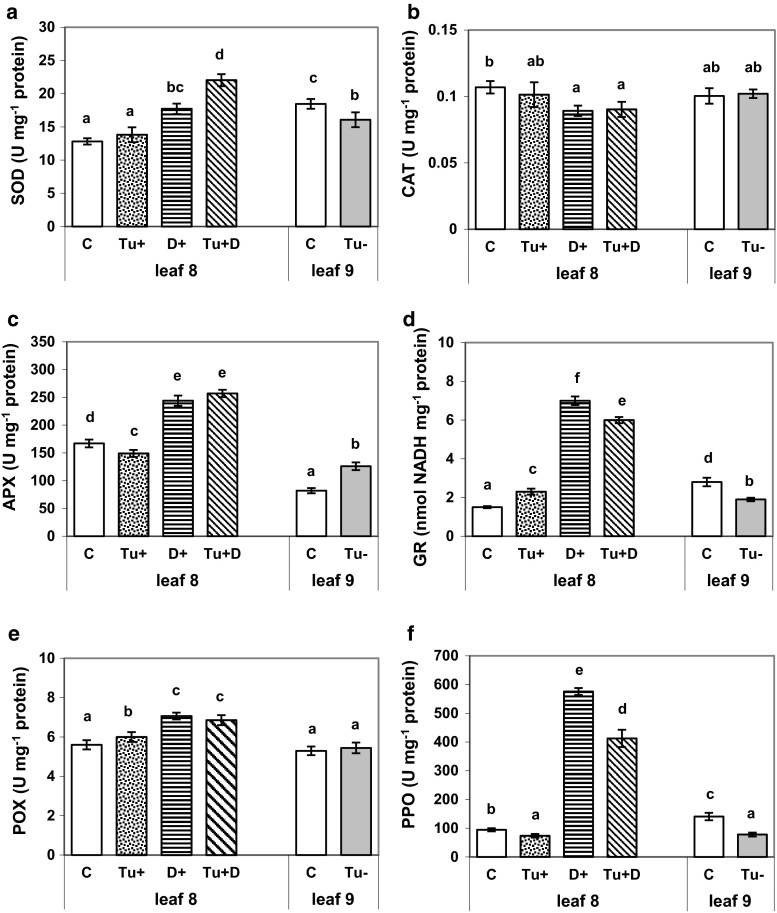
**a**–**f** Superoxide dismutase (SOD, **a**), catalase (CAT, **b**), ascorbate peroxidase (APX, **c**), glutathione reductase (GR, **d**), guaiacol peroxidase (POX, **e**) and polyphenol oxidase (PPO, **f**) activity in maize leaf 8 grown under optimal control conditions (C), subjected to mite infestation (Tu+), soil drought (D+) and both stresses (Tu + D) simultaneously, and in noninfested leaf 9 (Tu−) in the immediate vicinity of mite-infested leaf 8 and respective control (C). *Different letters* above *bars* indicate statistically significant differences at the significance level 0.05 (**a**, **b**, **c**, **f**—HSD Tukey test; **d**, **e**—Kruskal–Wallis test)

RWC in maize leaf 8 subjected to mite infestation for 6 days decreased by less than 5 %, while at the same time, the soil drought occurring alone and simultaneously with the mite infestation, caused a reduction of the leaf RWC by 46 and 48 %, respectively. Exposing the experimental maize plants to the soil drought and mite infestation overlapping stresses (Tu + D) increased the activity of all the antioxidant enzymes in leaf 8, except for the CAT activity which markedly decreased (Fig. 1a–f). In contrast, the response of maize to mite infestation (Tu+) was not so uniform. In the mite-infested leaf 8, the SOD and CAT activity (Fig. 1a–b) remained unchanged while the APX and PPO activity (Fig. 1c–f) declined and the GR and POX activity increased (Fig. 1d–e). On the other hand, the 6-day water deficit (D+) caused by soil water shortage significantly increased the activity of all the antioxidant enzymes, except for CAT (Fig. 1a–f). The GR and PPO activity was markedly elevated by about 400 and 500 %, respectively. It is worth mentioning that although no mites were presented on leaf 9, the antioxidant enzymes became activated after leaf 8 had been mite-infested (Fig. 1a–f). However, the activity of antioxidant enzymes detected in mite-free leaf 9 changed in a different way, compared with the activity of antioxidant enzymes found in mite-infested leaf 8. For example, the APX activity increased by about 50 % (Fig. 1c) while the SOD and GR activity decreased by more than 13 and 30 % in mite-free leaf 9 (Fig. 1a, d). The CAT and POX activity did not change (Fig. 1b, e) while the PPO activity changed almost in the same way as in the case of mite-infested leaf 8 (Fig. 1f).

### Oxidative protein damage

**Fig. 2:**
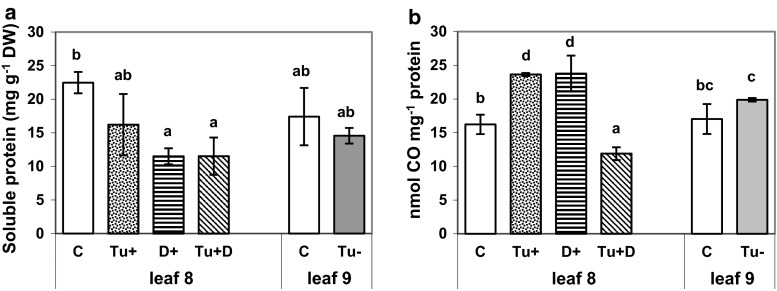
**a**–**b** Total protein content (**a**) and the concentration of derivatized carbonyl groups of oxidized proteins (**b**) in maize leaf 8 grown under optimal control conditions (C), subjected to mite infestation (Tu+), soil drought (D+) and both stresses (Tu + D) simultaneously, and in noninfested leaf 9 (Tu−) in the immediate vicinity of mite-infested leaf 8 and respective control (C). *Different letters* above *bars* indicate statistically significant differences at the significance level 0.05 (Kruskal–Wallis test)

Grown under optimal (well-watered) conditions, the mature maize leaves 8 and 9 were not differentiated by the soluble protein level (Fig. 2a). After mite feeding (Tu+), deleterious effect on the leaf 8 protein content was noted, while the soluble protein content decreased by about 50 % under the soil drought stress (D+) separately applied, and it remained at the same level under the combined drought and mite stresses (Tu + D).

To assess how much maize leaf proteins had been modified by various stresses, the protein carbonylation level was measured (Fig. 2b). The effect of soil drought and mite feeding applied together (Tu + D) differed from the one of a single stress. Both drought (D+) and mite feeding (Tu+) stresses applied separately elevated the protein carbonylation from 16.23 ± 1.44 to 23.64 ± 0.23 nmol C=O mg^−1^, while the combination of drought and mite feeding (Tu + D) decreased the content of carbonylated proteins by about 30 % to the protein level of 11.89 ± 0.95 nmol C=O mg^−1^. The carbonylated protein content in maize leaf 9 after being subjected to a 6-day mite feeding period on leaf 8 was the same as the carbonylated protein content of control leaf 9.

### Leaf proteomic changes

**Fig. 3:**
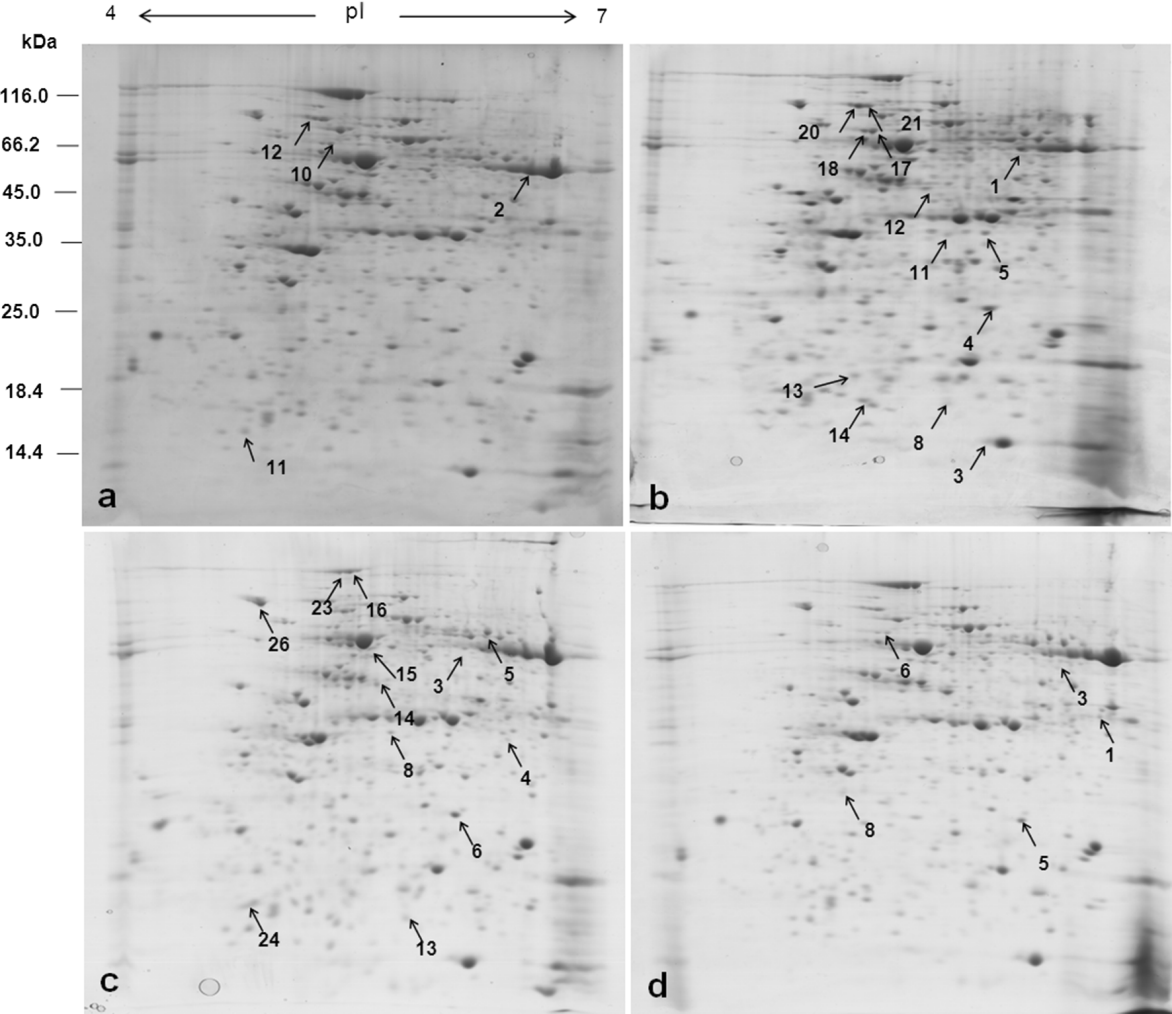
**a**–**d** Representative 2-DE gels of CBB-stained proteins extracted from mite-infested leaf 8 (**a**), soil drought-treated (**b**), double-stressed (**c**), and from noninfested leaf 9 close to the mite-infested leaf 8 (**d**). Within each gel, *numbers* indicating protein spots differentiating treatment and unstressed control correspond to those in Table 2 and 3. In the first dimension, IPG strips (Bio-Rad Laboratories, USA) of pH 4–7 (indicating p*I*) were used to separate proteins. In the second dimension, 11 % polyacrylamide gel was used. Standard molecular masses (kDa) are indicated

**Fig. 4:**
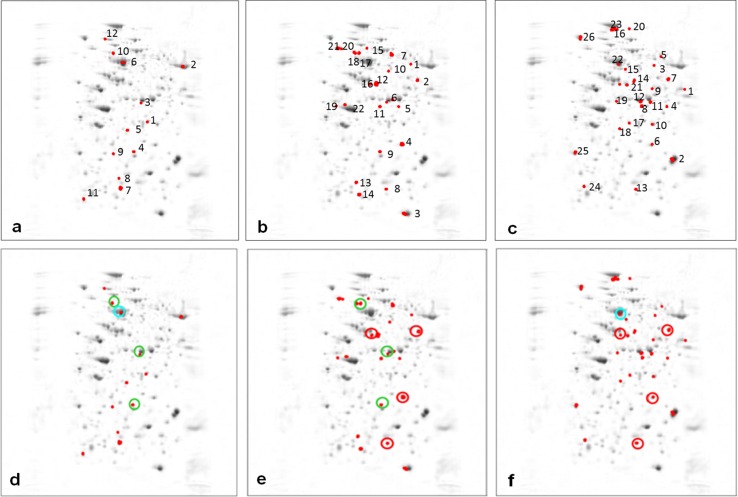
**a**–**f** Representative mean images with marked significant spots differentiating the classes—C(8) and [Tu + (8)] (**a**), C(8) and [D + (8)] (**b**) and C(8) and [Tu + D(8)] (**c**). Below, mean images presenting the spots shared by mite [Tu + (8)] and drought [D + (8)] stresses (marked green; **d** and **e**), by drought [D + (8)] and double stresses [Tu + D(8)] (*marked red*; **e** and **f**) and by mite [Tu + (8)] and double stresses [Tu + D(8)] (*marked blue*; **d** and **f**)

The representative 2-DE gel images of maize leaf proteins with the protein spots separately numbered for each treatment are shown in Fig. 3a–d. The results of PERMANOVA (Zerzucha et al. 2012) performed for 358 protein spots, summarised in Table 1, indicate that only four out of six pairs of the compared classes are statistically different (at significance level 0.05). As each analysis performed on the spots has serious limitations (mainly due to the fact that the spots overlapped), the analysis was performed on a pixel level as well, and this time all the compared classes of samples were statistically different at the significance level of 0.05 (Table 1). Therefore, the further data analysis and feature selection were performed on the pixel level. Using the UVE-PLS method, a set of pixels significantly differentiating each pair of the compared classes was identified and assigned to the corresponding spots (from now on referred to as ‘significant spots’). For leaf 8 samples, the significant spots differentiating the studied class pairs were marked on the mean image in Fig. 4a–c. There were 12 spots differentiating the control [C(8)] and the mite-infested leaf 8 [Tu + (8)] (Fig. 4 a), 22 spots differentiating the control [C(8)] and the drought-stressed leaf 8 [D + (8)] (Fig. 4 b) and 26 spots differentiating the control [C(8)] and the simultaneously stressed leaf 8 [Tu + D(8)] (Fig. 4 c). Three significant spots (marked green) were shared by mite infestation and soil drought stresses (Figs. 4 d, e). Of all the significant spots representing the effect of soil drought stress, four spots (marked red, Fig. 4e) were also shared by both stresses (Figs. 4f) while one spot (marked blue) was shared by the single mite and both stresses (Fig. 4d, f).

**Fig. 5:**
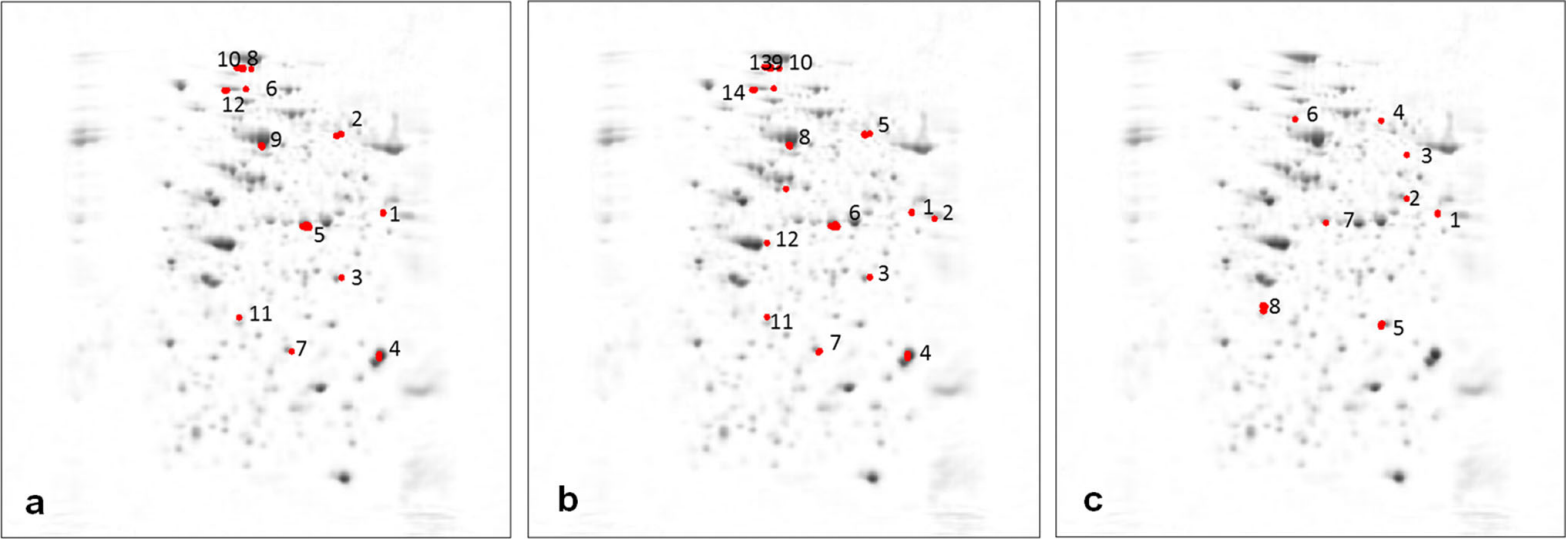
**a**–**c** Representative mean images with *marked* significant spots differentiating classes C(8) and C(9) (**a**), C(9) and Tu − (9) (**b**), Tu + (8) and Tu − (9) (**c**)

When studying the proteomic profiles of leaf 9, the following classes were compared: [C(8)] and [C(9)], [C(9)] and [Tu − (9)] as well as [Tu + (8)] and [Tu − (9)]. The identified significant spots (12, 14 and 8) marked on the mean image are presented in Fig. 5a–c, respectively.

While doing a multivariate discriminant analysis, it is possible to identify the features which are significant for classes discrimination but which do not individually differentiate the studied classes of samples (a univariate approach). As far as our study is concerned, of all the 12 spots differentiating the C(8) and Tu + (8) classes in the multivariate analysis, only four spots were significantly different when compared individually, while of all the 22 spots differentiating the C(8) and D + (8) classes only 13 were significantly different (Table 2). Similarly, of all the 26 spots differentiating the C(8) and Tu + D(8) classes only 12 spots were significantly different. Additionally, there were 12, 14 and 8 spots differentiating classes C(8) and C(9), C(9) and Tu−(9) as well as Tu + (8) and Tu − (9), however, the number of spots significantly different (when compared individually) was much lower: 4, 5 and 5, respectively (Table 2). Therefore, only those individually different protein spots were further subjected to the LC–MS/MS and then they were compared with the nonredundant protein database of NCBI.

**Fig. 6:**
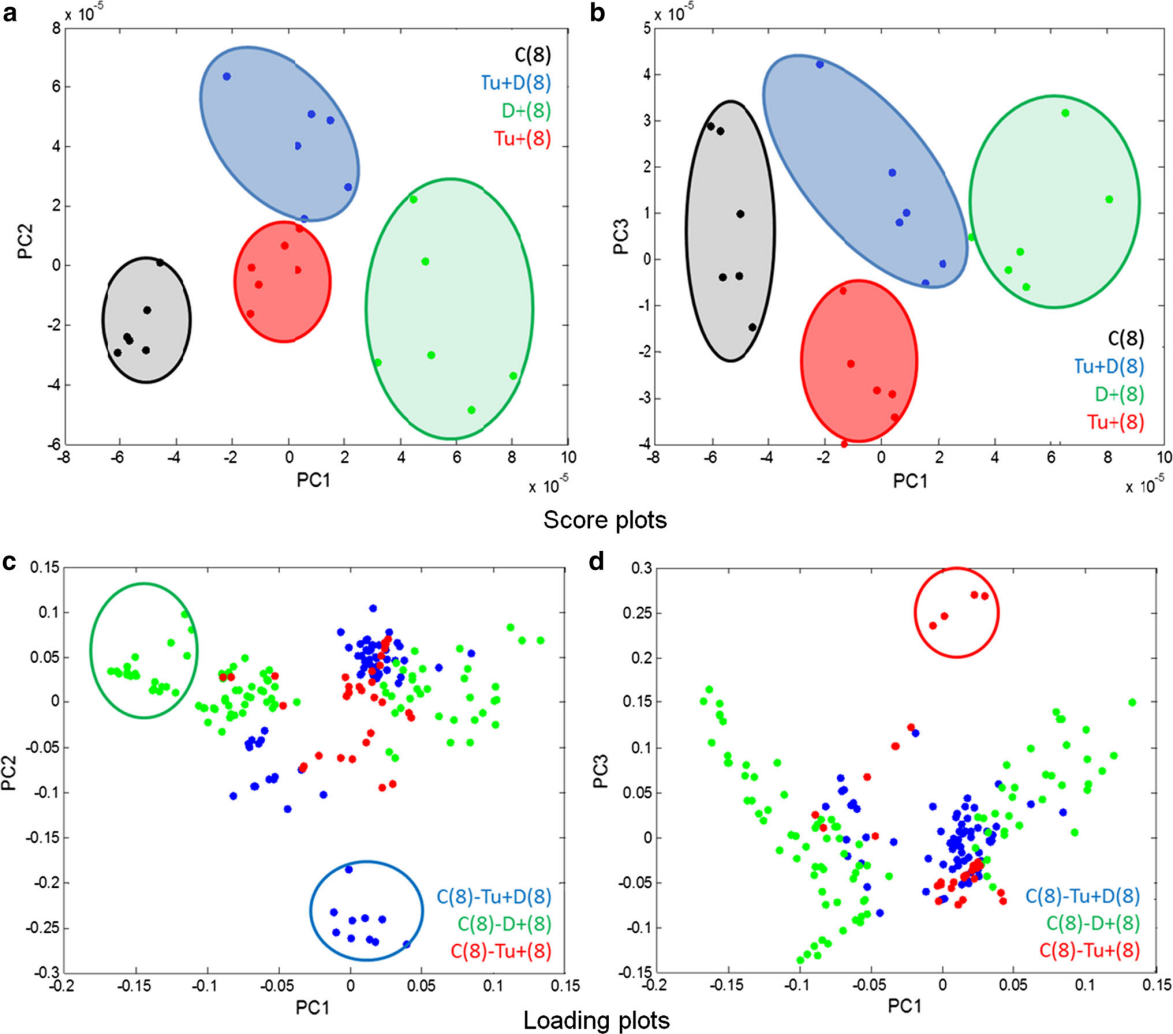
**a**–**d** The results from PCA of 24 samples obtained from four classes C(8), Tu + (8), Tu + D(8) and D + (8) presented in the form of score (**a**, **b**) and loading plots (**c**, **d**) onto the planes defined by PC1 and PC2, and PC1 and PC3, respectively

Using the results from the PCA, a certain insight into the biotic/abiotic stress impacts on the leaf proteome can be gained. To reveal the differences between leaf 8 samples, a PCA was performed on the (centred) data matrix containing all the features identified as significantly differentiating the classes of samples with the induced effect(s) (mite, soil drought, and the combination of soil drought and mite stresses) from the control one. The principal components (PCs) were constructed as linear combinations of the original features to maximize the description of data variance. The PCA made it possible to compress the obtained data into a few orthogonal hidden factors (PCs) and to visualise them in the low dimensionality space defined by PCs. The results from PCA are presented in the form of score and loading plots, representing projections of samples and features (pixels) onto the planes defined by the respective PCs. Score plots of 24 leaf 8 samples drawn on the planes and defined by PC1 and PC2, and PC1 and PC3, respectively, are presented in Fig. 6a–d. Looking at the previously discussed data set, the first three PCs describe 36, 20 and 10 % data variance, respectively. Each sample is represented by a point. If the points are close to each other, they have similar proteomic profiles. If they are apart, their proteomic profiles differ to a high degree. These projections reveal that the biggest difference in proteomic profiles, observed along PC1, is between the control class [C(8)] and the soil drought class [D + (8)] (Fig. 6a). Classes Tu + (8) and Tu + D(8) have similar coordinates on PC1 (close to zero) (Fig. 6a). PC2 reveals the difference of class Tu + D(8) from all the remaining classes (Fig. 6a), whereas the difference between class Tu + (8) and all the remaining classes is observed along PC3 (Fig. 6b). The PCA result proves that the soil drought stress influences protein profiles to the highest degree, whereas a combined effect of the soil drought and mite infestation has a relatively weaker effect on maize leaf 8 proteome. The corresponding loading plots presented in Figs. 6c–d, allow identifying the features (pixels) responsible for the observed sample patterns. The pixels within the green, blue and red cycles contribute to PC1, PC2 and PC3, respectively, to the highest degree.

**Fig. 7:**
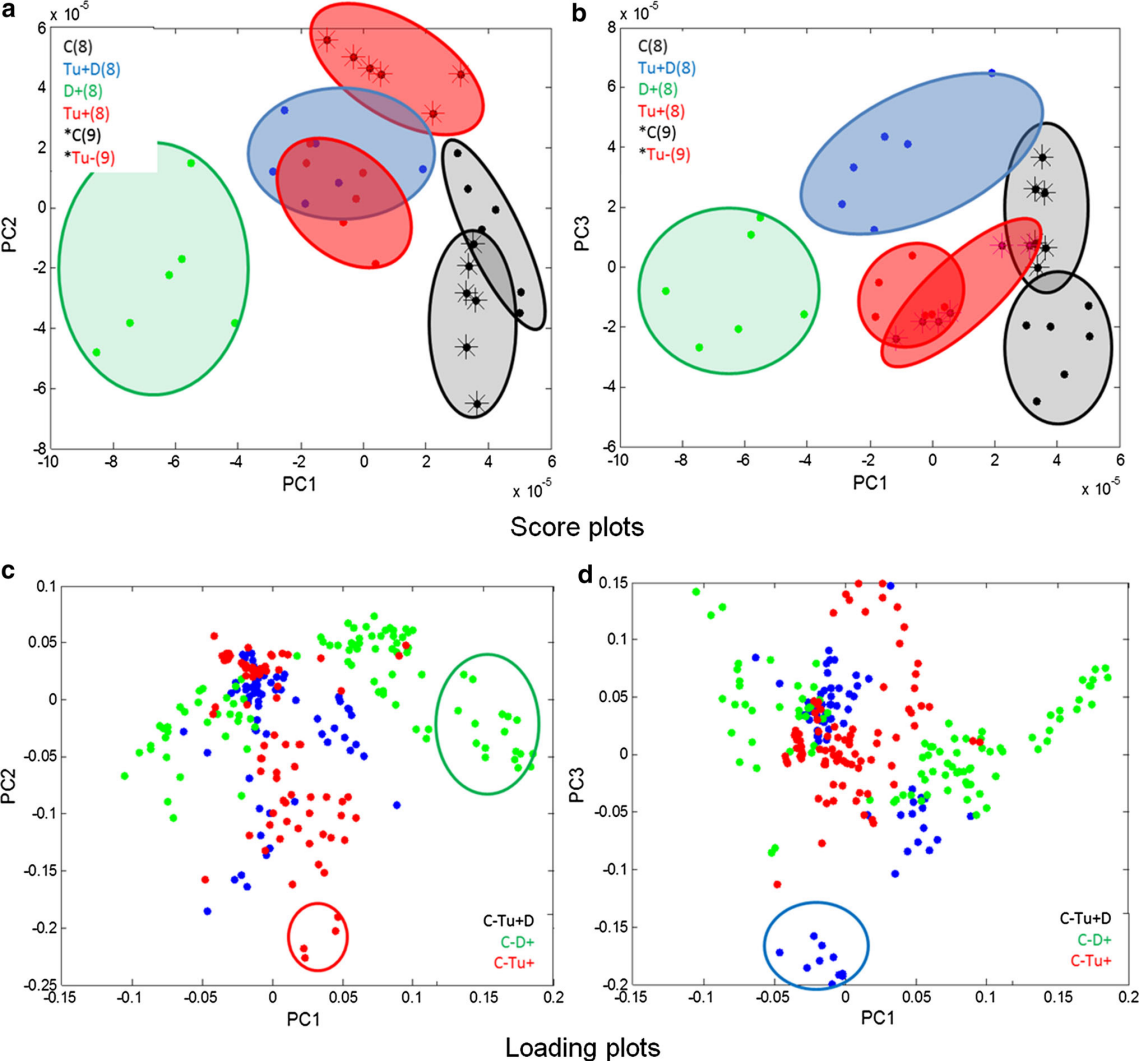
**a**–**d** The results from PCA of 36 samples obtained from six classes C(8), Tu + (8), Tu + D(8), D + (8), C(9) and Tu − (9) presented in form of score (**a**, **b**) and loading plots (**c**, **d**) onto the planes defined by PC1 and PC2, and PC1 and PC3, respectively

**Fig. 8:**
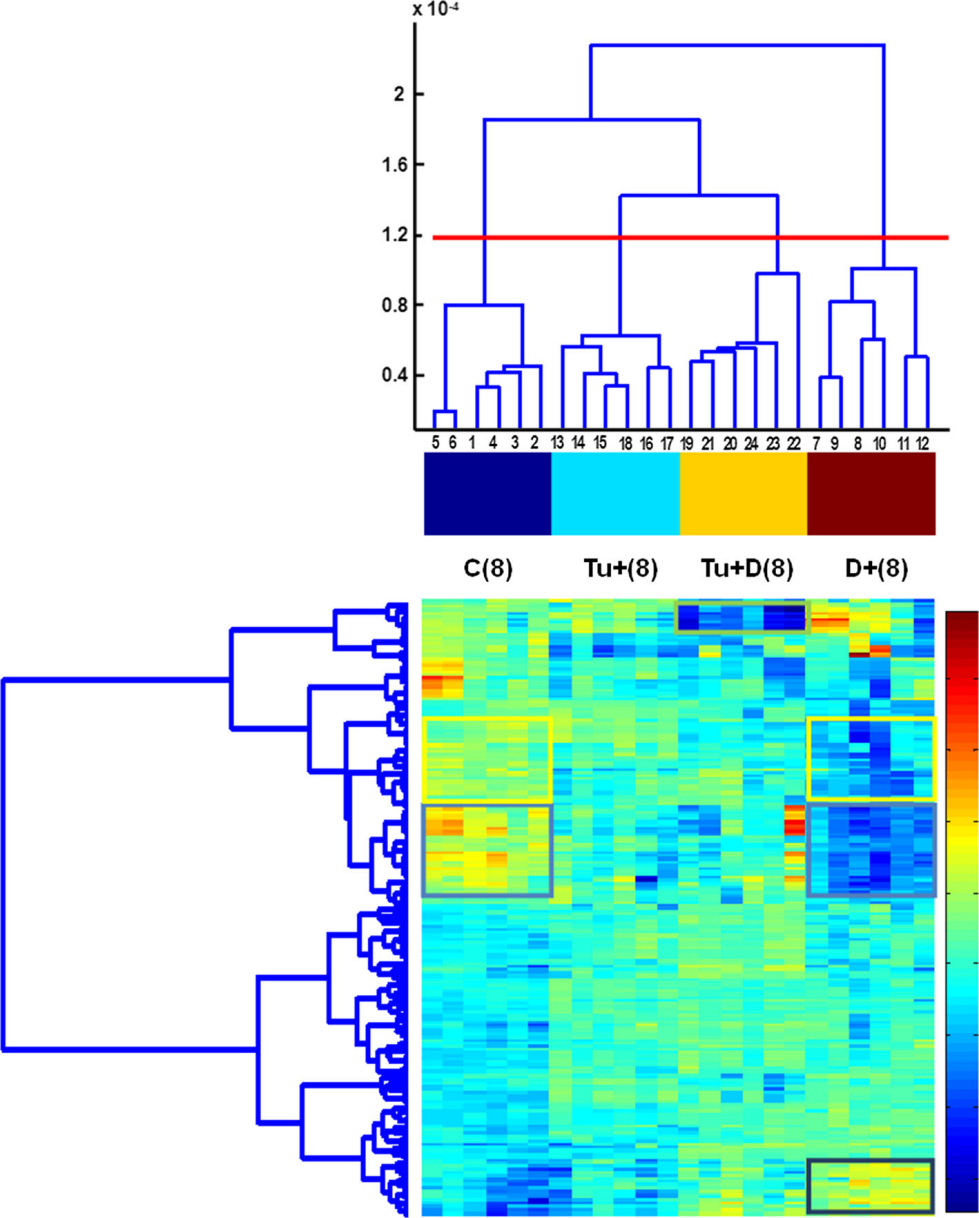
Dendrograms for 24 samples obtained from four classes [C(8), Tu + (8), Tu + D(8), D + (8)] augmented with the heat map of centred data matrix (with columns and rows sorted out in the corresponding dendrograms). Gradations of colour from *dark blue* to *red* in the *colour bar* indicate the increase in value of data elements

**Fig. 9:**
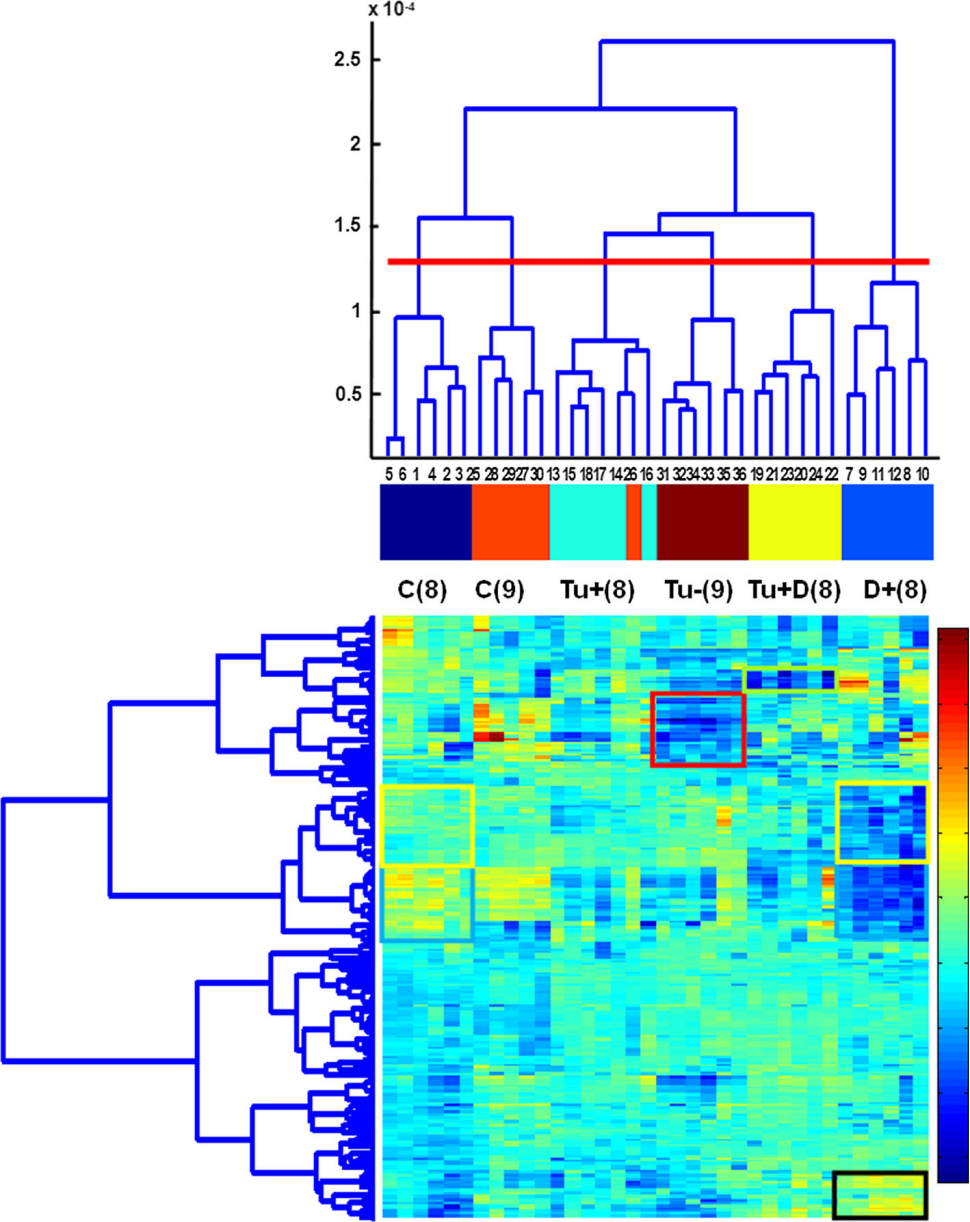
Dendrograms for 36 samples obtained from six classes [C(8), Tu + (8), Tu + D(8), D + (8), C(9), Tu − (9)] augmented with the heat map of centred data matrix (with columns and rows sorted out in the corresponding dendrograms). Gradations of colour from *dark blue* to *red* in the *colour bar* indicate the increase in value of data elements

The PCA score plots of all the six studied classes, i.e., of the 36 both leaf 8 and 9 samples, are presented in Fig. 7a–d. As shown, PC1 has not differentiated between leaf 8 and 9 (Fig. 7a–b). The differences between leaf 8 and 9 are mainly revealed by PC2 (and PC3, but to certain degree though). PC2 describes the differences between the C(9) and Tu − (9) classes (Fig. 7a). PC3 reveals the specificity of class Tu + D(8) (Fig. 7b). The D + (8) class samples display the greatest variance, whereas the remaining classes are more homogenous. The corresponding loading plots (Fig. 7c–d) revealed that the same pixels as in the case of the analysis of the 24 samples are responsible for designing the observed pattern of the 36 samples. It should be stressed that the patterns revealed in the PCA score plots represent the 66 and 72 % data variance only for 24 and 36 samples, respectively. Taking into account the total data variance, HC analysis was applied. The results of HC analysis are presented in the form of dendrograms. The indices of the clustered objects (or variables) are displayed on axis *x* of the dendrograms, whereas axis y represents the corresponding similarity measure between the two merging objects or clusters. Dendrograms obtained for the data sets containing 24 leaf 8 samples and 36 leaf 8 and 9 samples are presented in Figs. 8 and 9. They are augmented with heat maps (Smoliński et al. 2002) representing transposed data matrices. The rows of the matrices represent pixels and the columns represent samples. Matrix columns are sorted out in the dendrograms of the above samples, whereas rows are sorted out in the dendrogram of pixels. The way the samples are clustered is based on the Euclidean distance, whereas the way the pixels are clustered is based on their correlation.

In the dendrogram of leaf 8 samples (Fig. 8), there are four main subgroups corresponding to the studied sample classes. Sub-clusters Tu + (8) and Tu + D(8) are more similar to each other than to the remaining sub-clusters. They are more similar to C(8) than to D + (8), i.e., the most dissimilar is class D + (8). The heat map indicates which pixels are responsible for which (previously observed) clustering pattern. The results of the clustering 36 samples (Fig. 9) are quite consistent with the corresponding PCA results (Fig. 7a–d). The observed 6 sub-clusters of the studied samples correspond well with the 6 studied classes (the exception is one sample from class C(9), which appears in the cluster of the Tu + (8) samples. The structure of the dendrogram reveals similarities between C(8) and C(9) and between Tu + (8) and Tu − (9). Class Tu + D(8) is more similar to the sub-cluster containing the samples from classes Tu + (8) and Tu − (9) than to the remaining classes. The most dissimilar is class D + (8). To sum up, short-term soil drought causes greater changes in the leaf proteome profile than mite infestation. When occurring simultaneously, joint stress leads to specific changes in the proteome profile.

### Proteins identified under single and combined stresses

Table 3 presents detailed information (protein accession number, identification scores, molecular mass and isoelectric points, etc.) concerning 43 protein spots identified by LC–MS/MS. However, four proteins remain unknown due to the lack of their database matches while two have not been fully characterized. Additionally, all the other information concerning identified proteins (peptide sequences and modification sites located in the selected peptides, peptide scores, charge, theoretical and expected molecular weights, retention time) is shown in Table S1 and https://dl.dropboxusercontent.com/u/24272155/widma.zip.

The proteomic analysis showed that in the mite-damaged leaf 8 [Tu + (8)], heat shock cognate 70 kDa protein2 (HSC70), characteristic for stress response, and oxygen evolving enhancer protein3 containing protein (OEE3), involved in the functioning of the photosystem II (PSII) complex, were increased in abundance, whereas the abundance of ribulose-bisphosphate carboxylase/oxygenase (RuBisCO; EC 4.1.1.39), a crucial contributor to the Calvin–Benson cycle, and putative TCP-1/cpn60 chaperonin family protein (cpn60) were decreased (Table 3). In response to soil water deficit [D + (8)] eight proteins in leaf 8 were increased in abundance (Table 3). Three of them, small and large RuBisCO subunits and NADP-malic enzyme (L-malate: NADP oxidoreductase, oxaloacetate decarboxylating, EC 1.1.1.40; NADP-ME) are related to photosynthesis; 17.5 kDa class II heat shock protein, cpn60 and LOC 100192117 (pathogenesis-related PR-10 protein) are defence/stress responsive; glyoxylase1 (lactoylglutathione lyase; EC 4.4.1.5) is involved in recycling the reduced glutathione (GSH) and maintaining glutathione homeostasis. Four of the identified proteins (i.e., drought-inducible 22 kDa protein, plastid ADP-glucose pyrophosphorylase large subunit (ADP-GlcPPase; EC 2.7.7.27), chloroplast protein synthesis2 (cps2), and LOC 100281701 (RuBisCO large subunit-binding protein subunit α) were decreased in abundance.

In leaf 8, in response to both mite feeding and soil drought stresses [Tu + D(8)] phosphoenolpyruvate carboxylase (PEPC; EC 4.1.1.31), three isoforms of pyruvate orthophosphate (Pi) dikinase (PPDK; EC 2.7.9.1), precursor of β-d-glucosidase (EC 3.2.1.21), drought-inducible 22 kD protein, aspartate aminotransferase (AAT; EC 2.6.1.1) and stromal 70 kDa heat shock-related protein were found to be increased in abundance (Table 3). The expression of putative peptidyl-prolyl *cis*–*trans* isomerase family protein isoform1 (PPIase; EC 5.2.1.8) and cps2 was decreased.

**Fig. 10:**
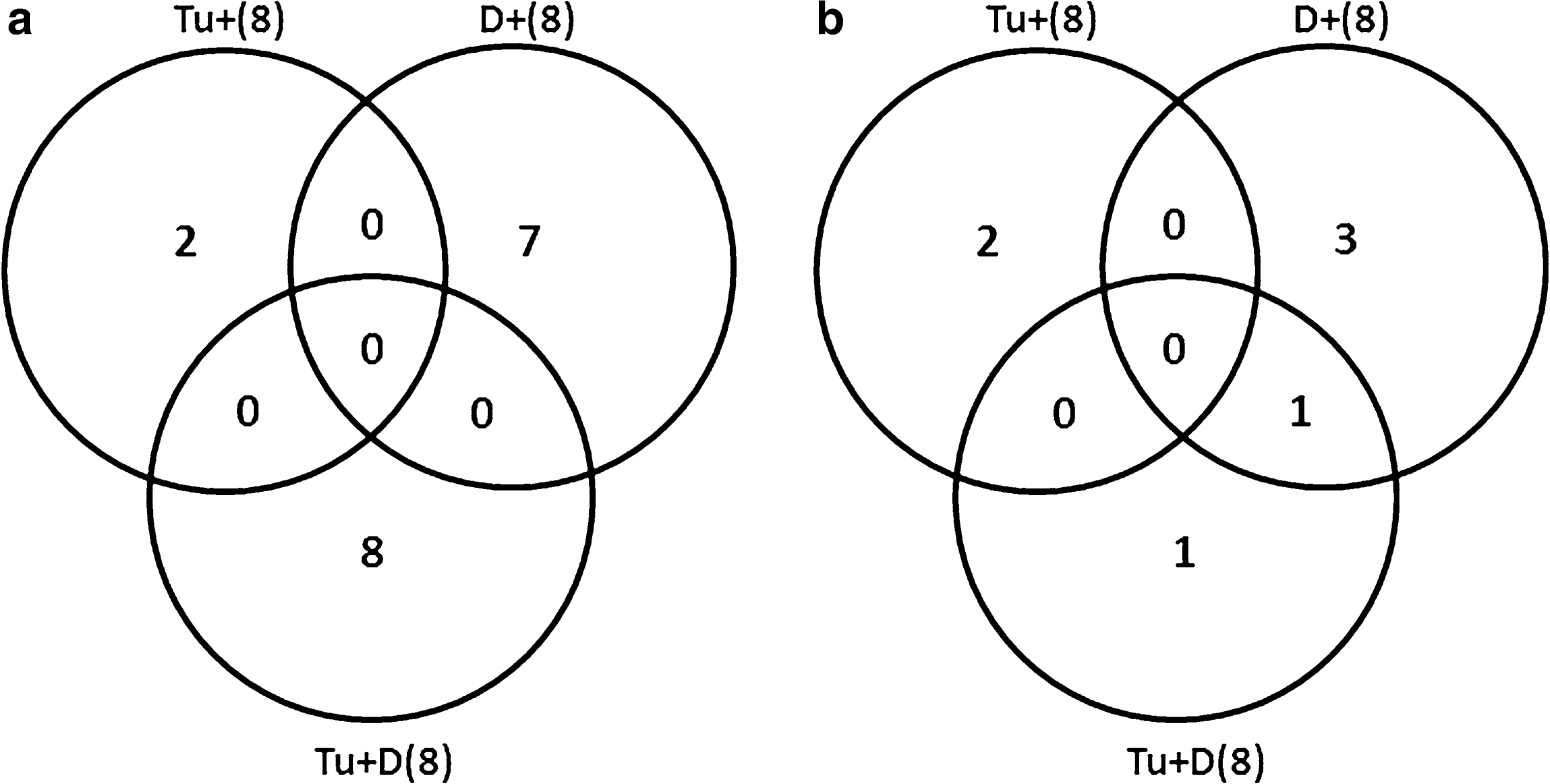
Venn diagrams showing the overlapping of increased (**a**) or decreased (**b**) abundance of maize leaf proteins upon mite infestation [Tu + (8)], soil drought [(D + (8)] and a combination of stresses [Tu + D(8)]

In summary, Venn diagrams (Fig. 10) show that of all maize leaf 8 proteins that increased in abundance, none were found to be shared by the tested classes [Tu + (8); D + (8); Tu + D(8)], whereas of all the proteins that decreased in abundance, only cps2 was affected by the D + (8) and Tu + D(8) stresses.

The comparison of leaf 8 [C(8)] with leaf 9 [C(9)] protein profiles shows that in leaf 9, which was younger than leaf 8, ATP synthase CF1 α subunit (atpA; EC 3.6.3.14) was increased in abundance while pyruvate phosphate dikinase (PPDK) and two other proteins involved in glycolysis [fructose-bisphosphate aldolase (EC 4.1.2.13), glyceraldehyde-3-phosphate dehydrogenase (GAPDH; EC 1.2.1.12) were decreased in abundance (Table 3). Similarly, the comparison of noninfested leaf 9 [Tu − (9)] from mite-infested plants with leaf 9 from control plants [C(9)] reveals that atpA was increased in abundance, while PPDK, two GAPDH isoforms and fructose-bisphosphate aldolase were decreased. In the mite undamaged leaf 9 [Tu − (9)] above the mite-damaged leaf 8 [Tu + (8)], the abundance of five proteins was systemically changed (Table 3). The abundance of RuBisCO large subunit-binding protein subunit β, fructose-bisphosphate aldolase and superoxide dismutase [Mn] 3.4, mitochondrial precursor (Mn-SOD; EC 1.15.1.1) was increased, whereas the expression of fructose-bisphosphate aldolase and GAPDH was decreased.

## Discussion

### Oxidative stress-related enzymes and soluble proteins

Our study shows that soil water deficiency and mite feeding stresses, when imposed individually, increased the guaiacol POX and GR activities and diminished the CAT activity in maize leaf 8. Similarly, CAT-2 isoform (the main contributor engaged in the removal of photorespiratory H_2_O_2_) decreased in both mite-infested maize and citrus leaf tissues (Maserti et al. 2011; Świątek et al. 2014) as well as in drought-stressed non-Bt maize (Świątek et al. 2014). Under drought, the APX was more responsive than the CAT. It suggests that intracellular H_2_O_2_ level could be controlled by CAT-independent pathways (Mhamdi et al. 2010; Brossa et al. 2015). The rise in the GR activities under individually applied drought and mite stresses implies enhanced regeneration of reduced glutathione (GSH) from oxidized glutathione (GSSG) in the ascorbate–glutathione cycle (Foyer and Noctor 2011). The observed here changes in the activity of GR were more pronounced under drought than mite stress. Interestingly, the combined effects of these two stresses resulted in the decreased GR activity, whereas the POX and APX activities remained at the level noted for soil drought stress. The increase of POX activity when maize leaf has been affected by both mite and drought stresses, acting separately or together, seems to confirm the enzyme involvement in the plant defensive processes (e.g., ROS metabolism regulation, lignin/suberin formation, cross-linking of cell wall polymers, hypersensitive responses, etc.) as in the case of other plant species either infested with phytophagous mites (Stout et al. 1996; Kielkiewicz 2002) or subjected to soil drought (Lee et al. 2007).

PPO is involved in plant defence against various stresses, including soil drought (Mayer 2006) and mite infestation (Duffey and Felton 1991; Stout et al. 1996; Kielkiewicz 2002). PPO catalyses the oxidization of cell wall cross-linking phenolics and phenolic polymerization to highly reactive quinones, that may convert amino acids into antinutritive compounds for herbivorous pests (Duffey and Felton 1991). The enhanced PPO activity in the dehydrated maize leaf 8 on one hand, and the inhibited one in the mite-infested leaf on the other, which was observed in this study, suggest that the PPO responds differently to each of the individual stresses. However, it is not quite clear whether strong stimulation of the PPO activity is beneficial or detrimental to drought-stressed plants (Mayer 2006). In comparison to the effect of both stresses applied individually, the combined effect of soil drought and mite feeding stresses resulted in an increased activity of SOD and reduced activity of GR and PPO, suggesting distinct defence responses, which is in accordance with the current research (Prasch and Sonnewald 2015).

Finally, it is worth noting that in leaf 9 (free of mites and in close proximity to mite-infested leaf 8), the APX activity increase and the simultaneous decrease in the activity of SOD, GR and PPO, indicate the systemic effect of mite infestation, in which ascorbate–glutathione cycle enzymes and enzyme-oxidizing phenols are thought to be involved. Although there is an increasing evidence confirming antioxidant enzymes and phytohormones engagement in systemic responses monitoring biotic/abiotic tolerance (Zebelo and Maffei 2015; Xia et al. 2015), further research is needed for a full understanding of the phenomenon in the C4 monocot—mite interactions.

In this study, we observed that soil drought stress drastically reduced the maize leaf hydration, but co-occurring mite infestation did not contribute to further leaf water content decrease. Similarly, soil drought or mite feeding, occurring individually, decreased the soluble protein content, while the combined stresses were not additive in this respect. The decline in the content of soluble proteins seems to have been caused by the intensified degradation of damaged or unnecessary proteins (Benešová et al. 2012). Inactivation or breakdown of proteins may also result from protein carbonylation, the major form of protein oxidation regarded as a marker for oxidative stress (Levine 2002). The dehydration-induced increase in both protein carbonylation and activity of antioxidant enzymes (SOD, APX, GR, POX, PPO), shown in the present paper, suggests that a 6-day soil water deficit resulted in protein oxidative damage in maize leaves. This is consistent with the decrease in the efficiency of photosystem (PS)II photochemistry (Fv/Fm), a widely used parameter to assess the photosynthetic apparatus functioning under stress conditions (Brossa et al. 2015), from 0.739 ± 0.066 in control leaf to 0.601 ± 0.063 in drought-stressed leaf (data not shown). However, the effect of mite feeding stress on the induction of leaf oxidative stress is less evident. In mite-infested leaves, the increase in oxidative carbonylation coincided with the reduced CAT, APX and PPO activity at a constant Fv/Fm (0.772 ± 0.015 as compared to 0.739 ± 0.066 for control leaves). Surprisingly, under both stresses, protein carbonylation decreased despite the increased activity of all antioxidant enzymes (except the CAT activity) and Fv/Fm decreased from 0.739 ± 0.066 to 0.592 ± 0.073 (data not shown). In light of our data, protein carbonylation is not directly linked to oxidative stress based on the assessment of ROS enzymatic scavengers. Protein carbonylation may also be a result of diminished capacity of oxidized protein removal, increased protein susceptibility to oxidative attack or other unknown yet interrelations. It should be underlined that the determination of carbonylated proteins points only on the type of posttranslational protein modification, but protein network modification under simultaneously applied biotic/abiotic stresses remains unknown.

### Maize leaf proteome

To the best of our knowledge, the proteome analysis was not previously carried out to reveal the differences in the defensive responses of commercial maize to environmental stresses, such as mite infestation and soil drought, applied either individually or in combination. A multivariate analysis (UVE-PLS) allowed to identify 94 protein spots (out of 358 considered) which differentiated the studied treatments. Only 43 of them had individual discrimination power, and they were positively identified by searching across protein database of NCBI-NR and grouped by their biological relevance. Upon mite feeding, the abundance of RuBisCO that fix CO_2_ in Calvin–Benson cycle decreased in maize leaf 8, as it was previously observed in rice on which the brown planthopper (*Nilaparvata lugens* Stål) fed, in *Nicotiana attenuate* plants on which the *Manduca sexta* caterpillars fed, in *Solanum tuberosum* L. plants infested with the Colorado potato beetle (*Leptinotarsa decemlineata* Say) larvae or in tomatoes challenged by the potato aphid (*Macrosiphum euphorbiae* Thomas) (Giri et al. 2006; Wei et al. 2009; Duceppe et al. 2012; Coppola et al. 2013). Conversely, RuBisCO increased in abundance in citrus leaves on which the two-spotted spider mite fed (Maserti et al. 2011). One of putative reasons of decreased RuBisCO abundance seems to be a coincidental lowered abundance of Cpn60, a recently discovered molecular chaperone responsible for RuBisCO folding and assembly (Trösch et al. 2015).

OEE3 and heat shock cognate 70 kDa (HSC70) protein2 were induced upon mite feeding. OEE3 is known as one of three proteins forming the oxygen evolving complex (OEC), which maintains the manganese cluster of the PSII complex in a chloroplast. Therefore, it can be reasoned that in mite-infested leaf tissue, OEE3-enhanced abundance improved the light-capturing ability protecting the leaf against photoinhibition, as it has been shown in the maize and soybean leaves exposed to short-term mite injuries (De Freitas Bueno et al. 2009). The increased amount of HSC70 protein2, one of the stress-inducible heat shock protein (HSP70) homologs which exhibits low constitutive expression, indicates its involvement in stabilising the nascent proteins released from ribosomes, thus protecting the partially synthesized polypeptides from being accidentally misfolded or aggregated (Zhu et al. 2012).

In the drought-stressed maize leaf 8, 13 protein spots differed in their expression pattern, and among them, the small and large RuBisCO subunits were increased in abundance, similarly as in the acclimated wheat, barley and sugarcane exposed to soil water deficiency (Zhou et al. 2012; Shanker et al. 2014). Since Cpn60 is uniquely important for RuBisCO folding and assembly (Trösch et al. 2015), its coincidental increase in the dehydrated maize leaf 8 indicates that soil drought did not affect RuBisCO itself. Drought stress-induced abundance of NADP-ME (another photosynthesis-related protein) seems to maintain the rate of the RuBisCO-catalyzed reaction in maize leaf 8. It was confirmed that NADP-ME activity in rice increased under salt, osmotic and drought stress (Ke et al. 2009), and as NADP-ME was overexpressed, it improved salt and osmotic tolerance in *Arabidopsis* (Liu et al. 2007) and tobacco (Laporte et al. 2002). It is also well documented that under stress conditions, the reducing power (NADPH) produced by NADP-ME mediated L-malate decarboxylation is used for ROS detoxification (Laporte et al. 2002). The drought-induced abundance of stress-related glyoxylase1, one of two enzymes of the glyoxalase system, which is the major pathway of metabolism of methylglyoxal (MG) in the cytosol and mitochondria, illustrates the effectiveness of cytotoxic MG and other 2-oxoaldehydes conversion into 2-hydroxyacids, using GSH as a cofactor, in an irreversible two-step reaction. This point to glyoxylase1 engagement into plant tolerance and oxidative defence against soil drought and other abiotic stresses (Zadražnik et al. 2013).

Considering the function of sHSPs-like molecular chaperones that bind partially denatured proteins, one may suppose that, in dehydrated maize leaf 8, the increase in abundance of 17.5 kDa class II HSP prevents from irreversible protein aggregation. Similarly, overexpression of LOC100192117 (pathogenesis-related PR-10 protein) suggests its protective function (Liu and Ekramoddoullah 2006). Among proteins that decreased in abundance following maize leaf 8 dehydration, ADP-GlcPPase, cps2, RuBisCO large subunit-binding protein subunit α and drought-inducible 22 kDa were identified. The lowered abundance of ADP-GlcPPase suggests that the starch biosynthesis declined because the product (ADP-Glc) of the ADP-GlcPPase catalyzed reaction is the major substrate for starch biosynthesis in photosynthetic and non-photosynthetic tissues. The limited cps2 expression indicates disorders in the translation process of chloroplast proteins because this protein participates in driving the translation machinery (Belcher et al. 2015). RuBisCO large subunit-binding protein subunit α binds RuBisCO small and large subunits and is involved in the assembly of the enzyme oligomer (Hauser et al. 2015). Its decreased abundance, together with the decreased abundance of cps2, gives an indication of drought-induced impairment of protein biosynthesis. The lowered expression of 22 kDa protein implies that it was not involved in improving maize tolerance to short-term water deficit. Nevertheless, 22 kDa protein contributes to acclimation of sugarcane seedlings to osmotic stress (Zhou et al. 2012) and to maize defence against pathogens (Huang et al. 2009).

We documented that the proteome response of maize leaf 8 to the combined mite and drought stresses significantly differed from those induced by each stress applied individually, which is consistent with current research (Atkinson and Urwin 2012; Atkinson et al. 2013; Prasch and Sonnewald 2015). The combined stresses result in the increased abundance of PEPC, PPDK isoforms, AAT, as well as the proteins with potential protective functions (β-d-glucosidase precursor, predicted stromal 70 kDa heat shock-related protein, drought-inducible 22 kDa protein). We therefore assume that these proteins are responsible for maize adjustment to novel environmental conditions. PEPC (one of the essential cytosolic enzyme in the C4 photosynthesis) was induced by soil drought, salt and cold (Doubnerová and Ryšlavá 2011), but nothing is known about PEPC involvement in response to mite-pest infestation. In a transgenic maize line, higher drought tolerance was related to PEPC overexpression (Jeanneau et al. 2002), but in *Sorghum bicolor* genotypes, it was not (Jedmowski et al. 2013). The increased abundance of another protein—PPDK (catalysing the formation of phosphoenolpyruvate, PEP) in maize leaf 8 by combined stresses is in full agreement with PPDK up-regulation in drought tolerant genotypes of *Sorghum bicolor* (Jedmowski et al. 2013) and higher drought tolerance in rice (Gu et al. 2013). We are unable to indicate which form of PPDK was increased (the cytoplasmic or the chloroplastic). Despite that one may suppose that the PEPC and PPDKs increased abundance improves the efficiency of carbon fixation in maize leaf 8 under simultaneously applied soil drought and mite feeding stresses. Moreover, due to the elevated abundance of PEPC and PPDK, many metabolic pathways (including citric acid cycle or amino acid synthesis) should be provided by an increased level of intermediates. This suggestion is in accordance with the increased abundance of AAT that may result in greater availability of aspartate to biosynthesis of the aspartate-family amino acids (methionine, lysine, asparagine). In *Arabidopsis*, under the combined soil drought and nematode attack, the methionine metabolism was intensified, resulting in higher tolerance to the pest infestation (Atkinson et al. 2013).

β-d-glucosidase abundance, which increased in maize leaf 8 under soil drought and mite feeding acting as one, suggests the contribution of the enzyme to support plant defence. However, the elevated activity of β-d-glucosidase is indicative of intensified lignification, phytohormones production from Glc-conjugates or plant defending activity against biotic/abiotic stresses (Morant et al. 2008). β-d-glucosidase has also been shown to activate DIMBOA, a secondary maize metabolite that is toxic to chewing and phloem-feeding herbivores (Pentzold et al. 2014).

Under combined drought and mite stresses, a maize plant defended itself by increasing the expression of HSP70-class molecular chaperones. The enhanced expression of HSP70 should protect plants against stress-induced improper folding/refolding polypeptides and facilitate degradation of unstable proteins, protein aggregate solubilisation, protein complexes disassembly and control of membrane translocation (Gupta and Tuteja 2011; Trösch et al. 2015 and rfs therein). When the stresses overlapped, the drought-inducible 22 kDa protein was also found to have been raised in maize leaf 8. However, it decreased in a dehydrated maize leaf. This clearly shows that unlike the separately applied soil drought stress, the mite feeding stress truly induces the expression of drought-inducible 22 kDa protein when maize is under double stress.

Among the proteins decreased in abundance under coexisting stresses, there were cps2 (decreased by water deficit as well), peptidyl-prolyl cis–trans isomerase (PPIase) and an unknown protein. PPIase is one of two foldases known to be involved in folding and trafficking proteins (Gupta and Tuteja 2011). White pine weevil-induced tissue of Sitka spruce (Lippert et al. 2007) showed to be overabundant in PPIase, whereas the reduced abundance of PPIase in the double stressed maize leaf 8 tissues seems to implicate the proper protein folding disruption.

AtpA, known as energy metabolism-related protein, was found to be overabundant in the noninfested leaf 9 of a mite-infested plant. This may imply a higher demand for ATP to improve stress tolerance in the noninfested leaf 9 of mite-infested maize plant. The abundance of PPDK, as well as glycolytic protein (fructose-bisphosphate aldolase and GAPDH) isoforms was decreased.

A comparison of proteome of the noninfested maize leaf 9 with proteome of the mite-infested leaf 8 revealed changed expressions of five proteins. RuBisCO large subunit-binding protein (subunit β), one of fructose-bisphosphate aldolase isoforms and mitochondrial precursor of Mn-SOD, belonged to proteins that increased in abundance, while another fructose-bisphosphate aldolase isoform and GAPDH were lowered in abundance. RuBisCO large subunit-binding protein (subunit β) belongs to the chaperonin (HSP60) family that binds RuBisCO small and large subunits and is involved in assembling the oligomer enzyme (Trösch et al. 2015). However, as we still do not know how systemically it works, the issue needs to be further investigated.

Fructose-bisphosphate aldolase exists in the chloroplastic and cytosolic isoforms, and in the noninfested leaf 9, it was identified as two spots. One of them decreased in abundance while the other one increased. This shows that fructose-bisphosphate aldolase is not only regulated by the abiotic stresses as it was shown previously (Houston et al. 2009), but also by the mite-pest stress and in a systemic way. The elevated abundance of Mn-SOD indicates that one of the predominant SOD isoforms responds systemically to biotic stress. Up to now, the enhancement of Mn-SOD activity was documented in citrus leaves on which mite fed locally (Maserti et al. 2011).

### Concluding remarks and perspectives

Taken together, multivariate chemometric methods for analysing proteomic data reveal that the changes in the maize leaf proteome under soil drought stress are greater than those under mite infestation stress or under both stresses combined. Nevertheless, a particular adjustment in the maize leaf proteome profile under separately or simultaneously applied stresses was documented. For example, it was shown that mite infestation decreases the abundance of leaf maize proteins related to photosynthesis, whereas soil drought increases it. In the mite-injured leaves, proteins which protect the photosynthetic apparatus against photoinhibition increase in abundance. Both stresses, when acting simultaneously, elevate the amount of proteins that enable maize to maintain the efficiency of photosynthesis and metabolism, as well as to protect its cells against metabolic injuries. It is noteworthy that soil drought co-occurring with mite infestation increases the abundance of photosynthesis-related proteins (PEPC, PPDK) located in the mesophyll cells, whereas soil drought acting individually increases the abundance of NADP-ME functioning in the bundle sheath cells. Consequently, PEPC, PPDK and NADP-ME can be differently modified to improve maize efficiency of CO_2_ fixation upon individual and double stresses.

The results presented in this study confirm that the overabundance of relevant proteins should be an adequate method to improve plant crop tolerance to joint stresses. Further analyses of the overexpressed/silenced monocot mutants will have to be made to explore the functions of proteins modified under individual/coexisting stresses in greater detail. The results of such research, together with applied proteomic approach, should significantly contribute to developing maize genotypes able to tolerate coexisting environmental stresses.

#### *Author contribution statement*

MK and BZ designed research and wrote this paper. MN conducted proteomic experiments. AD, MN, DS-Ł, AM carried out biochemical analyses. BW and MK analysed data. All authors participated in the analysis of this study and read the final version submitted.

## Electronic supplementary material

Below is the link to the electronic supplementary material.

Suppl. Fig. S1 a-b Pseudo-colour display of original images 1 and 2 from control class (C8) (before warping) with the correspondent spots marked (**a**), and the same images after warping (**b**) (TIFF 1281 kb)

Suppl. Fig. S2 a-c Mean image of the studied data set (**a**), identified protein spots (**b**) and binary mask (**c**), which allows data analysis at the pixel level (TIFF 199 kb)

Suppl. Table S1 The detailed technical data on the identified proteins (XLSX 53 kb)

## Abbreviations

APX: Ascorbate peroxidase
CAT: Catalase
GR: Glutathione reductase
PEPC: Phosphoenolpyruvate carboxylase
POX: Guaiacol peroxidase
PPDK: Pyruvate orthophosphate (Pi) dikinase
PPO: Polyphenol oxidase
ROS: Reactive oxygen species
SOD: Superoxide dismutase
