# Supplementary figures and images for: Maize proteomic responses to separate or overlapping soil drought and two-spotted spider mite stresses
Source: Planta. 2016 Jun 22;244(4):939–60. doi: 10.1007/s00425-016-2559-6 (PMC5018026; doi:10.1007/s00425-016-2559-6)

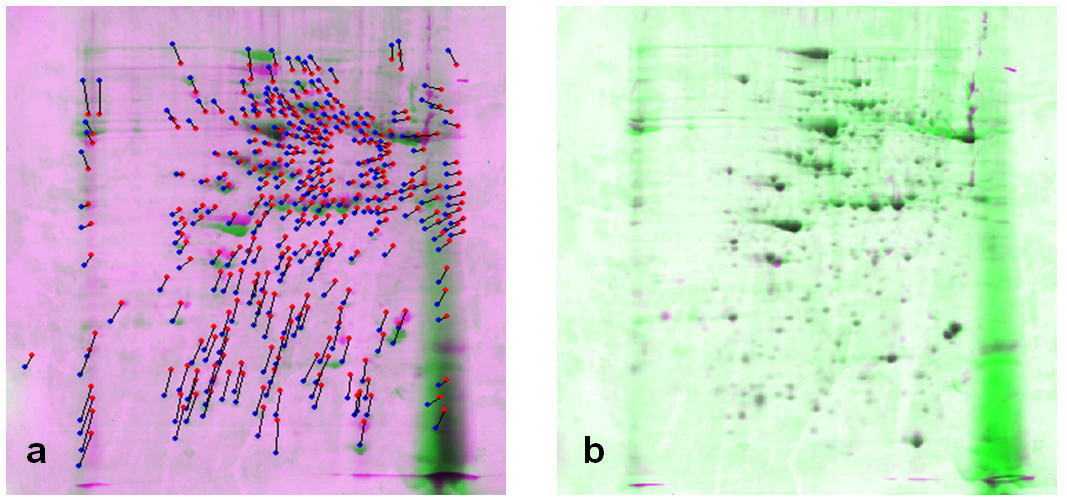

Supplement: Supplementary file 1 — Pseudo-colour display of original images 1 and 2 from control class (C8) (before warping) with the correspondent spots marked (a), and the same images after warping (b) (TIFF 1281 kb) [file 425_2016_2559_MOESM1_ESM.tiff]

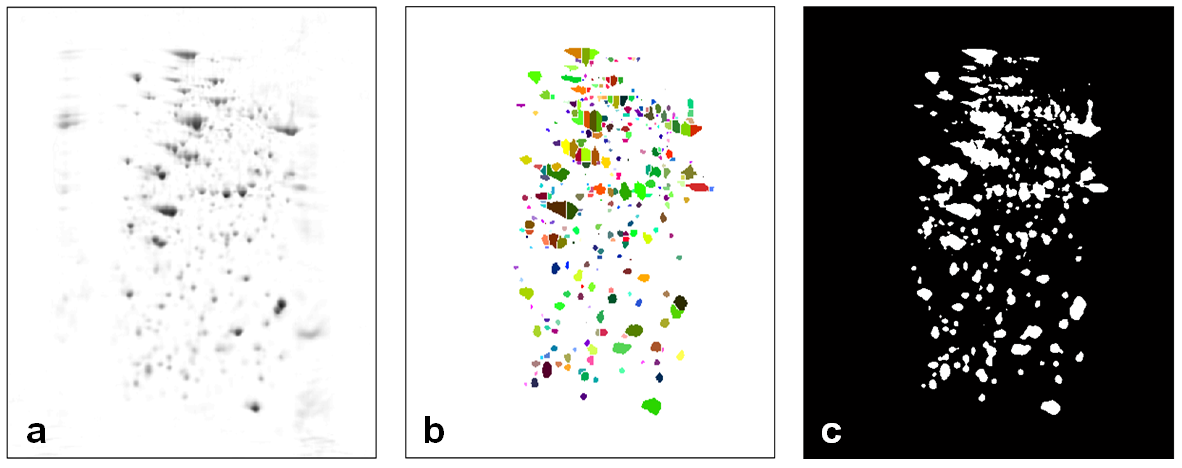

Supplement: Supplementary file 2 — Mean image of the studied data set (a), identified protein spots (b) and binary mask (c), which allows data analysis at the pixel level (TIFF 199 kb) [file 425_2016_2559_MOESM2_ESM.tiff]
